# Influence of Line Strategy Between Two Turns on Performance in Giant Slalom

**DOI:** 10.3389/fspor.2020.589257

**Published:** 2020-11-25

**Authors:** Clément Delhaye, Matthew R. Cross, Maximilien Bowen, Pierre Samozino, Frédérique Hintzy

**Affiliations:** ^1^Univ. Savoie Mont Blanc, Inter-University Laboratory of Human Movement Biology, Chambéry, France; ^2^Département Scientifique et Sportif, Fédération Française de Ski, Annecy, France

**Keywords:** alpine skiing, performance, trajectory, GNSS, force-plate

## Abstract

In alpine ski racing, different line choices can drastically affect turn or sectional performance. The straight-line transition between two turns is the main phase where skiers can gain speed in a race, open their trajectory, or reduce their path length. Between two turns, a skier can foster speed increase by spending more time in a straight line, inducing sharper turning phases (Z strategy). Inversely, speed can be conserved during the entire turn cycle by performing long curved turns separated by a short straight line (S strategy). This research aimed to evaluate the kinetic and kinematic specificities associated with the line strategy and to explore interactions of selected strategy with skier performance and energy dissipation. A mixed-level population of male alpine skiers (*n* = 17) skied a timed giant-slalom course while equipped with specialized force plates and a positional device collecting synchronized normal ground reaction force and position-time data, respectively. Time of edge switch was computed from the force signal as the period with the lowest force application on the outside ski. From positional data, turn cycles were separated into turning and straight-line phases (radius bellow and above 30 m, respectively). Time length, path length in the straight line, speed amplitude, and change in specific mechanical energy were computed for each turn and averaged for each skier. The path length during straight line was used to continuously characterize the line strategy within the spectrum between the Z (long straight line) and S (short straight line) strategy. Path length in the straight line was correlated with the amplitude of speed over a straight line (*r* = 0.672, *p* = 0.003) and relative and absolute time spent in the straight line *(r* = 0.967*, p* < 0.001). However, path length in straight line was not correlated with decrease of speed in the following turn (*r* = −0.418, *p* = 0.390) or time without force application on the outside ski (*r* = 0.195, *p* = 0.453). While higher-performing athletes on the course performed turns during which they dissipated less energy when normalized to entry speed (*r* = −0.620, *p* = 0.008), it appears they did so with variable turn strategies approaches.

## Introduction

To achieve the lowest possible race time, alpine skiers must continuously adapt their strategy and technique. Skiers perform turns according to their position, speed, and balance (influenced by the previous turn), individual capacity evolving with fatigue (technical, physical, psychological), and environmental factors (e.g., snow characteristics and micro reliefs, steepness, visibility, course setting, ski gear) (Supej and Holmberg, 2019). Some parameters cannot be anticipated before the race, which means skiers must continuously adapt their turn line strategy throughout the race.

In this article, the alpine skiing turn is defined by a straight line (SL) followed by a turning phase (TP). The trajectory and overarching turn characteristics the skiers choose (i.e., “line strategy”) can generate substantial differences in turn-time in giant slalom (GS) (Brodie et al., 2008) or on the following sections (Supej and Cernigoj, 2006). Skiers accelerate quasi-exclusively by converting potential energy into kinetic energy during the SL, while aerodynamic and ski/snow friction dissipate energy notably during TP. Energy dissipation characteristics are used to quantify performance (Hébert-Losier et al., 2014) and are commonly defined by the differential of the sum of kinetic and potential energy normalized to skier mass (m) (Supej, 2008; Reid et al., 2009). Since performance is partially dependent on the turns preceding it, normalizing energy dissipation to the velocity at the entry of the corresponding segment (*v*_in_) provides a means of characterizing sectional performance (Δ*e*_mech/_*v*_in_) (Supej and Holmberg, 2019). From a mechanical perspective, better turns are described by dissipating less energy via friction while following the most direct trajectory, which maximizes the transfer between potential to kinetic energy (Supej, 2008).

At every turn, skiers must balance between two strategic line approaches (Supej, 2008; Federolf, 2012).

The first line strategy focuses on reducing mechanical energy dissipation throughout the TP by avoiding skidding. This line strategy is typified by “carving” the skis in long arcing turning phases initiated higher on the slope and greater turn radii during the TP (Müller and Schwameder, 2003). It induces longer TP time, typically longer path lengths, an “S”-shape trajectory, and less speed gained during the SL due to less time spent close to the fall line. Barring high-energy dissipation during the TP, the technique should induce a low variation of energy dissipation during the turn. Coaches advise adopting this technique during low-dynamic phases and in flat, high-speed, and straight sections and do not recommend it after periods of high-speed dissipation (for example after heavy braking due to mistakes or cornering) (Lind and Sanders, 2004). The second-turn line strategy is based on maximizing the transfer of potential to kinetic energy between gates by following a trajectory closer to the fall line. Following this line strategy, skiers will typically delay the beginning of a TP and create a long SL between short TP resulting in a “Z”-shape trajectory. Since more time is spent close to the fall line, skiers gain more speed, and dissipate little energy during the extended SL phase. However, the approach is also characterized by sharper turns (i.e., lower radii), which might change ski–snow interactions and induce skidding and associated energy dissipation during the TP. Consequently, this approach is recommended by coaches in steep, turning, and high dynamic sections. Skiers adopting these two approaches could feasibly gain the same turn or sectional performance, with drastically different energy behaviors (i.e., variability in energy dissipation within the turn) during each turn. Theoretically, these two strategies are called “S” and “Z” strategies in this article, respectively, and represent opposite ends of a continuum of performance separated and identified by length of the SL.

Few studies have assessed the interaction between line strategy and performance. Articles focusing on turn line strategy and Δ*e*_mech_ in giant slalom (Supej, 2008) or in slalom (Federolf, 2012) have not presented clear correlations. This result may be linked to the small and homogeneous observed samples or specific factors relating to the course setup (e.g., a small number of gates observed). Moreover, for skiers with higher *v*_in_ (typical of high-level skiers), increased velocity or preserved energy is more challenging than for other skiers, and the normalization per entry velocity (i.e., Δ*e*_mech/_*v*_in_) effectively quantifies skiing efficiency. Consequently, subsequent investigations presented a very strong correlation between Δ*e*_mech/_*v*_in_ and race time in slalom (Supej et al., 2011) and in GS (Spörri et al., 2018). In the latter example, the authors presented a mechanical model of performance that quantified the weighted contribution of the capacity to gain or maintain speed relative to the previous section performance (i.e., Δ*e*_mech/_*v*_in_) and the trajectory of the skier (as the cumulated distance per turn) (Spörri et al., 2018). Better-performing skiers typically minimized energy dissipation relative to Δ*e*_mech/_*v*_in_, without an association with path length. The authors concluded that potentially the turn may be separated into phases with distinct aims, with the former half of the turn characterized by minimizing dissipation of energy and a shortening of the path length being preferable in the latter. Spörri et al. (2012) reported that a higher altitude of turn initiations and turn ends were associated with a reduction of time on short sections around the analyzed turn—albeit based on only one skier trial. However, while the interaction between line strategy and performance is associated with minimizing sectional energy dissipation relative to entry velocity, SL, and TP specific energy behaviors have never been clearly assessed.

According to Newtonian physics, a skier must apply external force to deviate from rectilinear motion to turn. When a skier is turning with carving technique, a radial force (i.e., in the direction of the center of the turn) is applied and depends on the speed, the mass of the skier, and the turn radius (radial force [*F*_*r* =_m·velocity^2^/*r]*). The radial force can be considered as the component of the ground reaction force acting in the direction of the center of the turn. Therefore, to increase this force, a skier can theoretically increase the lateral angulation with the snow and increase the resultant normal reaction force (i.e., the normal reaction force applied to the ski in the ski reference, nSkiRF). The positive correlation between mean nSkiRF value and performance is shown by some studies on direct and indirect measurement (Cross et al., 2019). Moreover, the application of nSkiRF differs with turn trajectory, radius (Nakazato et al., 2011), and skier level (Supej et al., 2011). According to the radial force equation above, at equal entry speed, the “Z”-shape trajectory strategy (i.e., associated with smaller turn radii during TP) will require higher nSkiRF during the TP, necessitating greater muscular output, and technical management of the ski snow contact (Federolf, 2012). High-level skiers present intrinsically better technical (i.e., capacity to carve at shorter turn radii) and physical skills (Vogt and Hoppeler, 2014; Franchi et al., 2019), which could allow them freedom to select the Z line strategy while maintaining ski snow contact with low skidding. The low variation of energy dissipation (i.e., “S” trajectory) technique is associated with carving, more extended turning phases, and longer force application time (Brodie et al., 2008); consequently, time to transfer force from one ski to the other could be shorter. This time without nSkiRF on the outside foot is considered as the edge switch moment and could be related to the line strategy used and the technical abilities of the skiers. In this case, technical abilities may allow skiers to perform complex tasks quicker, apply force more effectively to the snow, and continually adapt their trajectory. The assessed lag between force application and consequences on trajectory could be an interesting parameter of technical level. However, the interaction between force application and energy dissipation strategies has never been directly assessed.

The turn switch (TS) is the event used to separate two turns. Despite appearing to be a key event of strategic decisions for skiers, behavior during this period has attracted less attention in the research. At present, there are several ways to separate a course into individual turns via detection of the TS from kinematic and kinetic markers (Fasel and Gilgien, 2016) and subsequently separate a course into individual turns. In the first instance, the available methods are dictated by the sensors involved during the experimentation: stereoscopic 3D cameras (Supej and Nemec, 2003; Spörri et al., 2016), GNSS (global navigation satellite system) with IMU (inertial measurement unit) (Fasel et al., 2016), or force plate (Schaff et al., 1996; Nakazato et al., 2011). These various sensors detect one or multiple events between two turns. According to various definitions, several events can define the end of a turn, the beginning of the following turn, or the switch event between two turns. In chronological order, the turning radius passes above 30 m (1), the skier stops applying force on external foot (2, corresponding to the ski-edge release), the minimum summated nSkiRF over a given turn cycle (3), beginning of force application on subsequent external foot (4, ski-edge taking), and a turn radius below 30 m (5). The order of these events is stable regardless of the skiers and turns (Delhaye et al., 2019). The time when the turn radius is above 30 m is associated with the duration of the SL and period between Fbeg to Fend is considered as edge switch time. While not tested, the distance traveled by the skier with a radius above 30 m and time without force application will theoretically vary according to the line strategy used. Since the timing of these TS events is feasibly a result of different techniques, biomechanical mechanisms, and/or interaction between them, a better understanding of the timing and energy behaviors during this phase could provide insight into skier performance.

The first aim of the study was to (i) identify the kinetic and kinematic characteristics associated with the line strategy. We hypothesize that line Z strategy (characterize by longer path length in the straight line) is associated with (i.a) shorter path length in entire turn, (i.b) greater speed amplitude during the straight line and the turning phase, (i.c) more energy dissipated during the turning phase and entire turn, (i.d) longer time in the straight line normalized to the entire turn time, and (i.e) a longer time without force application between turns. The second aim of the study was to (ii) evaluate the interaction between selected strategy and performance level. We hypothesized (ii.a) that high-level skiers would be more inclined to use Z line strategy and (ii.b) line strategy would be associated with final race time.

## Materials and Methods

### Participants

Seventeen male alpine skiers participated in this study (mean ± SD: age 28.6 ± 9.3 years, height 179.7 ± 5.7 cm, weight 75.6 ± 9.3 kg). Skiers had mixed performance levels, consisting of recreational club level (not ranked by International Ski Federation [FIS]) to top-ranked current and previous elite world cup competitors (FIS points < 2). Each skier was informed of the content of the study and gave their written consent to participate. The experiment was conducted under the Declaration of Helsinki and was approved by the local ethics committee of the Université Savoie Mont-Blanc.

### Experimental Protocol and Data Collection

Following 2–3 familiarization trials of the course while being equipped with the various experimental technology, skiers performed one or two timed runs on a 16-gate giant slalom course. Skiers were instructed to ski as fast as possible while avoiding the risk of falling. The athletes were provided with instructions to prioritize finishing the race and within these constraints reduce the race time as much as possible. The course setting was initially performed by a national ski coach, per a 16-gate GS setup with an average distance of 23 m and an offset of 8 m on a 25° inclined groom slope. Setup was close to competition setup featuring irregularities while avoiding side-slope, jumps, and slope-break during turns. Landmark poles were placed close to the course outside of the grooming area, and gate positions were measured relative to them and to these poles in order to triangulate them at every course setting. Skiers used the same pair of race skis (SALOMON Lab X-Race—Annecy—France; radius: 30 m, length: 193 cm) and were equipped with the same model of ski boots (SALOMON XLAB 140+ WC—Annecy—France). Skiers used their own race suits and other FIS-approved race clothing and protective gear (e.g., helmet, goggles, back protectors).

Throughout the duration of experimentation (30 days), snow quality (hardness, humidity, and temperature) and environmental conditions (temperature, wind, and visibility) were observed. Testing took place during the first 3 h after the resort opening, the slope was groomed every night, and snow cleaning was performed between every run to limit the formation of micro-reliefs. Testing was performed only if the temperature during the preceding night was cold enough to freeze the snow. If these factors were not acceptable, experimentation was delayed until the conditions stabilized. Skiers were equipped with onboard validated force plates (Falda-Buscaiot et al., 2016). These force sensors (Sensix—Poitiers—France) 1400 gr per foot and raised the skier by 6 mm. The reaction force normal to the ski on the outside foot (nSkiRF) was amplified and recorded at 200 Hz. The power supply and acquisition gear were placed in a hip bag for a total weight of ~1 kg. Calibration to body weight was performed by lifting each foot successively while being fully equipped with the technology and equipment. The calibration procedure was performed on the same flat hard surface before the start of the race. Position and doppler speed were recorded by a synchronized GNSS/IMU unit (MacLloyd—Paris—France) with factory fusion computations recorded at ~10 Hz mounted on the hip. Race time was measured with a FIS approved laser cell system (Tag-Heuer—La Chaux-de-Fonds—Switzerland), which was used as the top level performance variable in the subsequent analyses (i.e., better performance = lesser course time).

### Data Processing

Only the best performance (fastest time) was used for analysis, with the first turns of the race containing skating steps excluded. All the data analyses were performed using Matlab version R2019a (MathWorks—Natick—USA). To synchronize GNSS/IMU with a force plate, skiers hit the snow with their skis before and after the race to create acceleration and nSkiRF peak. Using these acceleration and force peaks, all data from force plate and GNSS/IMU were interpolated to resample data at 200 Hz. The “makima” method was used to perform a smoothing interpolation (with less undulation than “spline”).

**Figure 1:**
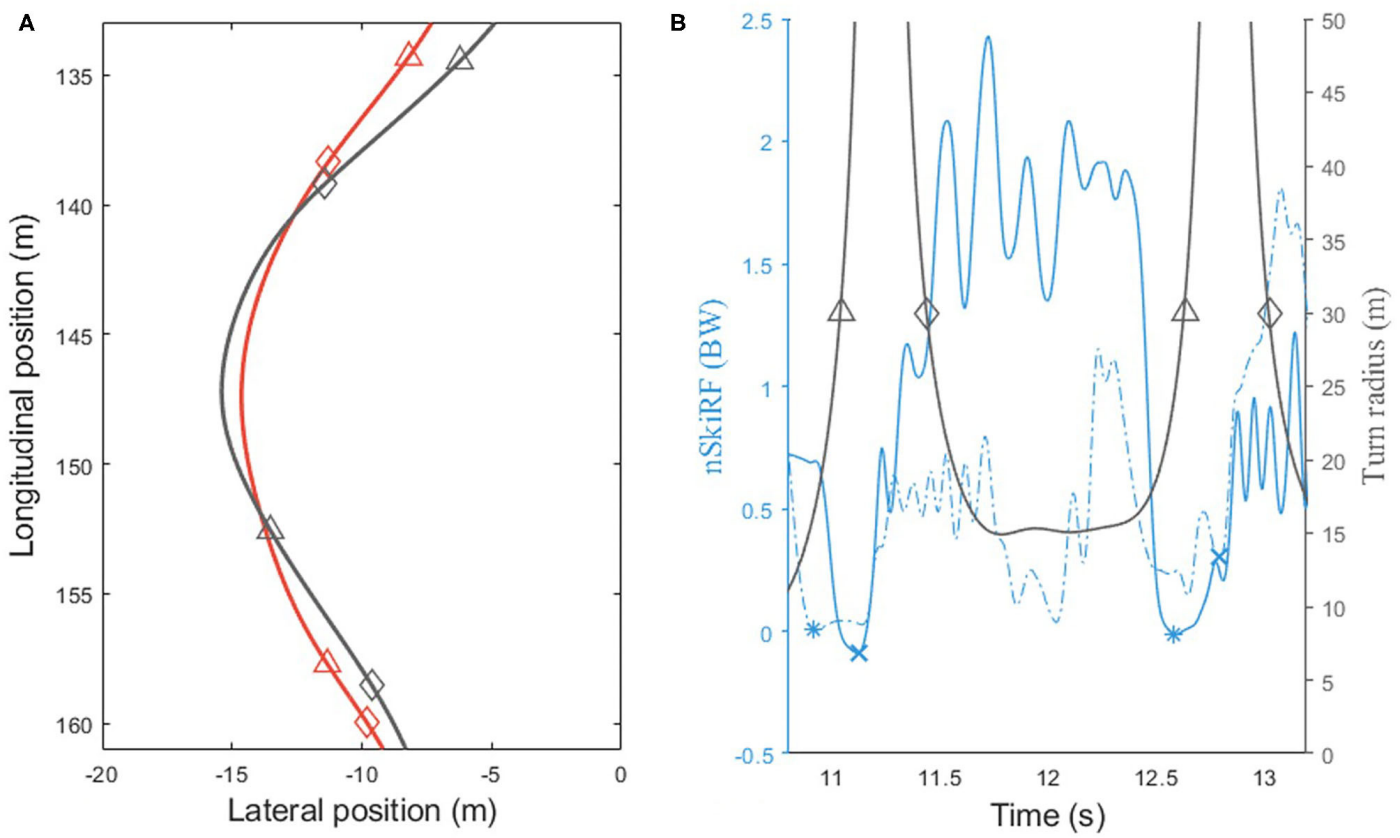
**(A)** Typical trajectory of skiers using Z line strategy (in gray) and S line strategy (in red). Triangles and diamonds correspond, respectively, to the turn radius passing above 30 m (Traj_>30_) and under 30 m (Traj_<30_), corresponding to the start and end of the straight line. **(B)** Typical evolution of the turn radius (in gray) and normal force applied to the ski (in blue) during a turn. Filled and dashed lines correspond, respectively, to the right and left foot; stars and crosses correspond to the end and beginning of force application.

**Figure 2:**
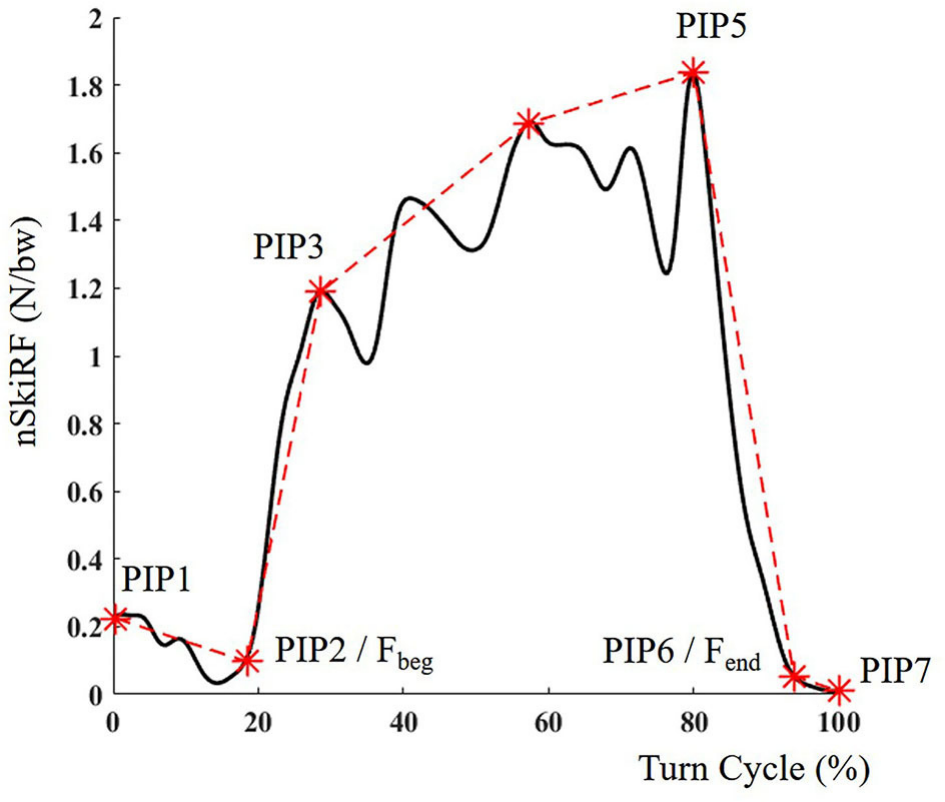
Typical detection of beginning (Fbeg) and end (Fend) of force application on outside foot nSkiRF signal (in body weight BW) during a turn expressed in percentage of turn cycle.

Four TS events were computed (Figure 1): two events from nSkiRF data and two from GNSS positional data. End of force application of the outside foot (F_end_) and beginning of force application on the outside foot (F_beg_) were detected on a 12-Hz low pass-filtered nSkiRF signal. The typical nSkiRF pattern on the outside ski during a turn is composed by a clear increase, a plateau, and a decrease (Figure 2); two consecutive patterns are separated by a period of low force application. Events on the force data were determined using the Perceptually Important Point method (PIP) (Fu et al., 2007). PIP is based on geometrical detection of the furthest vertical distance between data and a line between the endpoints of the interval. This computation is repeated five times to obtain the 7 PIPs (Figure 2). PIP6 and PIP2, respectively, represented F_end_ and F_beg_ events. All points were checked manually to ensure they corresponded to the end of the main decrease of force and the beginning of the main increase of force. The lag time between F_end_ and F_beg_ represents the switch between ski edges. The two events computed from positional data are the moment the turn radius passes above 30 meters (Traj_>30_) and under 30 m (Traj_<30_), corresponding to the end of the previous and start of the next turning phase, respectively (Spörri et al., 2012). Longitude and latitude positional data were converted into the Ellipsoid plane using WGS84 standard transformation. All radius and speed relevant parameters were computed using 2D data (longitude and latitude), and path length was computed in the 3D plane (longitude and latitude and altitude). Positional data were smoothed using a Savitzky–Golay filter (order 2, 1-s window). The radius was computed by circle fitting using the Pratt method (Pratt, 1987) (0.3-s window). Automatic detection was performed when the radius crossed the 30-m threshold, and in the case of multiple crossing at the TS last event was retained. The path length was computed as the cumulated displacement in all axes. The lag between Traj_>30_ and Traj_<30_ represents absolute time in SL (T SL in ms). Percentage of the turn in SL (T%SL in %) was defined as T SL relative to the sum of T SL and time of the following TP. The path length is computed for the SL and the sum of the SL and the following TP (Path SL+TP in m). Path length during edge switch was computed using the same approach (Path edge switch in m).

Speed was computed by the GNSS/IMU unit by Doppler measurement related to satellite motion. This computation method is more accurate than derivation of position. To compliment SL and edge switch detection, the amplitude of speed during the SL was computed as the difference between the minimum and maximum speed in the SL (ΔSpeed SL) and for the entire turn (ΔSpeed SL+TP).

Based on positional data, mechanical energy was computed at each point of the race as the sum of potential and kinetic energy, relative to the body mass of the skier (i.e., specific mechanical energy Supej, 2008; Supej et al., 2011). The change in specific mechanical energy was calculated for the following sectional divisions and normalized to the velocity of the first point (Δ*e*_mech/_*v*_in_): SL (Δ*e*_mech/_*v*_in_ SL), TP (Δ*e*_mech/_*v*_in_ TP), and SL + TP (Δ*e*_mech/_*v*_in_ SL+TP). Finally, race time is considered as the macroscopic indicator of skier performance level and Δ*e*_mech/_*v*_in_ SL+TPΔ*e*_mech/_*v*_in_ SL+TP is considered as the instantaneous performance level indicator.

### Statistical Analysis

Normality of each parameter was tested using the Shapiro–Wilk test except course time which approaches an expected flat distribution. To evaluate the first aim of the study (i.e., assess the kinetic and kinematic characteristics associated with the line strategy), the correlation between path length in SL and kinematic turn parameters (absolute time in SL, time ratio, speed amplitude in SL, speed amplitude in entire turn, path length in entire turn and Δ*e*_mech/_*v*_in_ in TP and during entire turn, time without force application) was tested using Pearson coefficient (*r*), the mean of skier turn cycles (*n* = 17). The second aim of the study regarding the interaction between line strategy and performance was also tested via correlation of straight-line length and race time (*n* = 17).

A hierarchical linear regression analysis was performed to determine whether the line strategy (assessed via the path length in SL) parameter improved the performance prediction provided by Δ*e*_mech/_*v*_in_ SL+TP. This parameter is already defined in previous literature as a predictor of performance and thus was first forced into the regression. The adding of path length in SL in the equation is tested in a second step to test its influence on race time prediction. Coefficient of determination (*r*^2^) and change in *r*^2^ (Δ*r*^2^) between models were calculated. All descriptive data were expressed as means ± standard deviation (SD), and all statistical analyses were performed on JASP (Version 0.13.1—Amsterdam—Netherlands) with an alpha level set at *p* = 0.05.

## Results

Mean course time was 29.47 s (SD: 1.85, min: 26.37, max: 34.61), the difference between the minimum and maximum time (i.e., 8.24 s). Once skating steps were removed, 185 turn cycles remained for the final analyses. Correlations between path length in the straight line and kinetics or kinematic parameters of the turn are reported in Table 1. T%SL (mean: 32.9%, SD: 4.32%, min: 26.78% max 43.58%) is well-correlated with the path length in SL (*r* = 0.967, *p* < 0.001).

**Table 1:** Correlation tests between path length in straight line (Path SL), and kinetic and kinematic parameters of the turn.

	***r* Pearson**	***p***
Path SL+TP (m)	−0.481	0.051
**ΔSpeed SL (m/s)**	**0.672**	**0.003**
ΔSpeed TP (m/s)	−0.418	0.095
Δe_mech/_v_in_ TP (J/kg/m/s)	−0.223	0.390
Δe_mech/_v_in_ SL+TP (J/kg/m/s)	−0.064	0.808
**T%SL (%)**	**0.967**	**<0.001**
**T SL (ms)**	**0.876**	**<0.001**
T Edge switch (ms)	0.195	0.453

*Path length in straight line is the cumulative distance traveled by the skiers between two turning phases where the radius is above 30 m. Successive correlated parameters are the path length in entire turn (both straight line and turning phase); speed amplitude during the straight line and turning phase; Δe_mech_/vin during the turning phase and entire turn; absolute time in a straight line, and relative time to the entire turn time. Edge switch time corresponds to the lag between end of the force application on the outside foot and start of the force application in the new outside foot and edge switch path to the distance travel during this period. Indices of correlation test Pearson's r (r) and p-value (p) were reported*.

**Figure 3:**
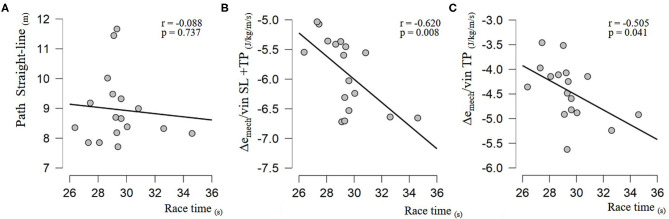
Correlation between race time (in seconds) and **(A)** path length in straight line (radius pass above 30 m), and **(B)** specific mechanical energy relative to entry velocity, respectively: during the turning phase (radius below 30 m), and **(C)** during the entire turn (from the beginning of force application on the outside ski to next one).

Secondly, mean path length SL is computed for each skier during the race. The mean path length in SL of the population was 8.96 m (SD: 1.16, min: 7.71, max: 11.67). Correlations between race time and path length in SL (*n* = 17) are presented in Figure 3A. Correlations between race time and specific mechanical energy dissipation (*n* = 17) are presented in Figures 3B,C. There was a moderate relationship of Δ*e*_mech/_*v*_in_ during turning phase (*r* = −0.505, *p* = 0.041) and entire turn (*r* = −0.620, *p* = 0.008) with race time.

The results of the hierarchical linear regression are summarized in Table 2. To assess the predictive power of the model, *F* and *F* change values were computed; the root mean square error (*RMSE*) of the predictive model (i.e., square root of the differences between observed and predicted race time values using full equation) was calculated to evaluate the accuracy of predicted race time in comparison with Δ*e*_mech/_*v*_in_ SL+TP alone; the *p-*value indicates the significance of the model prediction. Δ*e*_mech/_*v*_in_ SL+TP was forced in *Model 0*, path length in SL was added in *Model 1*, and path length in SL was finally not included in the model as a significant improvement of race time prediction (*p* = 0.301, *R*^2^ *change* = 0.047).

**Table 2:** Hierarchical linear regression model result, specific mechanical energy relative to entry velocity in entire turn (Δ*e*_mech/_*v*_in_ SL+TP) was forced in the *Model 0*; in *Model 1*, path length in SL was added to *Model 0*.

**Model**	***R***	***R*^**2**^**	**Adjusted *R*^**2**^**	**RMSE**	***R*^2^ change**	***F* change**	***F***	***p***
**Model summary of regression analyses to determine prediction of race time**								
0	0.621	0.385	0.344	1.566	0.385	9.396	9.396	0.008
1	0.657	0.432	0.351	1.558	0.047	1.152	5.321	0.301

## Discussion

Our first result focuses on the links between line strategy and mechanical or kinematic parameters of the turn. Line strategy has to be considered as a continuum characterized by path length in SL. However, the extremes of this continuum are dependent on the course setting and observed population. Consequently, this method only allows characterization of skiers on the same course. The increase in path length in SL was positively linked with longer absolute time in SL (*r* = 0.966, *p* < 0.001), and ΔSpeed SL (*r* = 0.554, *p* < 0.001). There was a trend for a negative correlation between the total path length during the entire turn and path length in SL (*r* = −0.481, *p* = 0.051). These results follow previous works (Federolf, 2012) and corroborate the statement that Z strategy is used by skiers to increase their speed between turns and decrease their total path length. However, a decrease of speed in the following turn was not correlated with the path length in SL (*r* = −0.418, *p* = 0.095). Skiers using the Z line strategy do not necessarily lose their additional speed gained in the SL in the following TP. Absence of significant results could be explained by the capacity of some skiers to conserve their speed even when using a Z line strategy. This capacity could come from a better technical level and better manipulation of the ski angle to decrease friction (Reid et al., 2020). This interpretation is supported by the absence of a relationship between strategy adopted and energy dissipation during the TP (*r* = −0.223, *p* = 0.390). Another explanation could be that some skiers using the S line strategy possibly drift at the beginning of the TP (Müller et al., 1998), which increases delta of specific mechanical energy even with a short path length in SL. This behavior is used when skiers exceed their “velocity barrier” (Supej et al., 2011), in other words when skier's speed is too high to perform the turn imposed by the gate setting.

Absolute and relative time in SL were strongly associated with the path length in SL. However, time of edge switch based on the nSkiRF measurements (i.e., the time between end and start of force application on the outside foot) was independent of the path length in SL. The time of edge switch was not influenced by temporal pressure variation induced by shorter or longer SL. Consequently, the time of edge switch should not be used to quantify the duration of the straight-line trajectory. Rather than turn line strategy, this parameter might be indicative of balance or technical abilities.

The main questions of this study concerned the link between the performance level and the line strategy. In the first instance, path length in SL is not correlated with Δ*e*_mech/_*v*_in_ SL+TP during entire turn (*r* = −0.064, *p* = 0.808). Moreover, a lack of relationship between path length in SL and race time (Figures 3A,B) indicates that the use of one predominant strategy is not necessarily linked with overall course performance. Absence of precise results is mainly due to the high variability of the strategy used by medium- and high-level skiers. Among skiers of a similar level (i.e., ranked 5th to 13th with race time between 28 and 30 s), there was substantial variation in SL path length. For example, two skiers performed mean SL path length above 11 m, yet others presented values <8 m. The choice of the strategy used could be determined by various anthropometrical (Haymes and Dickinson, 1980), physiological (White and Johnson, 1991), and technical profiles (Raschner et al., 1995; Müller et al., 2017). However, these interactions have not been recently evaluated on adult skiers.

Finally, according to linear regression results, Δ*e*_mech/_*v*_in_ SL+TP is negatively correlated with the performance, without a clear contribution of path length in SL. In agreement with previous works (Spörri et al., 2018), line strategy is not a predictor of the performance and does not lead to a decrease in the residuals of Δ*e*_mech/_*v*_in_ SL+TP correlation. As such, it would seem that independent of the line strategy used, reducing sectional energy dissipation is a key behavior to decreasing race time. These results highlight the presence of skier profiles able to decrease their dissipation of specific mechanical energy, in particular on a given strategy.

In conclusion, the strategy used is associated with differences in kinetic parameters. Increasing the path length in SL (related to Z strategy) induces a higher speed variation during SL, longer turn absolute and relative time spent in SL, and a tendency of shorter length path. However, the strategy used is not linked to time without force application on outside ski between two turns. Overall, the strategy adopted does not appear to be strictly associated with course-level performance of the skiers. The significant dispersion of strategies for the same performance level highlights that radically different approaches can result in similar energy dissipation characteristics. Consequently, it would seem that better skiers possess a profile that enables them to decrease energy dissipation across a variety of preferred and adopted turn strategies.

## Limitations

The top-level performance variable in this study was course time, which represents a macroscopic index and lacks the descriptive information potentially provided by more detailed analyses. Indeed, this parameter is not representative of each turn performance; a high-level skier who made a mistake is considered as the same level as a lower-level skier who did not. Moreover, race time is influenced by snow condition, and even under “acceptable snow conditions,” the snow quality variation between subjects could create differences between them. At last, race time is influenced here by the capacity of the skiers to be accustomed to ski gear and experiment devices. Finally, only male skiers were tested, and while we assume our findings apply to female skiers, more research is needed on female skiers.

## Data Availability Statement

The raw data supporting the conclusions of this article will be made available by the authors, without undue reservation.

## Ethics Statement

Each skier was informed of the content of the study and gave their written consent to participate. The experiment was conducted under the Declaration of Helsinki and was approved by the local ethics committee of the Université Savoie Mont-Blanc.

## Author Contributions

CD, FH, and MC conducted the data collection. CD, FH, MC, and PS conceptualized the study design and interpretation of the data. CD, MB, and MC contributed to the data analysis. CD drafted the manuscript. All authors revised it critically, approved the final version, and agreed to be accountable for all aspects of this work.

## Conflict of Interest

The authors declare that the research was conducted in the absence of any commercial or financial relationships that could be construed as a potential conflict of interest.
